# Evaluating the safety of early surgery for ruptured intracranial aneurysms in patients with long-term aspirin use: a propensity score matching study

**DOI:** 10.1186/s41016-020-00216-y

**Published:** 2020-11-30

**Authors:** Maogui Li, Shuzhe Yang, Qingyuan Liu, Rui Guo, Jun Wu, Yong Cao, Shuo Wang

**Affiliations:** 1 Department of Neurosurgery, Beijing Tiantan Hospital, Capital Medical University, 119 South Fourth Ring Road West, Fengtai District, Beijing, 100070 China; 2 China National Clinical Research Center for Neurological Diseases, Beijing, China; 3 Center of Stroke, Beijing Institute for Brain Disorders, Beijing, China; 4 Beijing Key Laboratory of Translational Medicine for Cerebrovascular Diseases, Beijing, China

**Keywords:** Intracranial aneurysm, Subarachnoid hemorrhage, Early surgery, Aspirin, Antiplatelet

## Abstract

**Background:**

Early microsurgical clipping is recommended for ruptured intracranial aneurysms to prevent rebleeding. However, dilemma frequently occurs when managing patients with current acetylsalicylic acid (aspirin) use. This study aimed to examine whether aspirin use was associated with worse outcomes after early surgery for aneurysmal subarachnoid hemorrhage (aSAH).

**Methods:**

We retrieved a consecutive series of 215 patients undergoing early microsurgical clipping within 72 h after aneurysmal rupture from 2012 to 2018 in the neurosurgery department of Beijing Tiantan Hospital. The medical records of each case were reviewed. Twenty-one patients had a history of long-term aspirin use before the onset of aSAH, and 194 patients did not. To reduce confounding bias, propensity score matching (PSM) was performed to balance some characteristics of the two groups. The intraoperative blood loss, postoperative hemorrhagic events, postoperative hospital stay, and functional outcome at discharge were compared between aspirin and non-aspirin group.

**Results:**

We matched all the 21 patients in aspirin group with 42 patients in non-aspirin group (1:2). Potential confounding factors were corrected between the two groups by PSM. No hospital mortality occurred after surgery. No significant differences were found in intraoperative blood loss (*P* = 0.540), postoperative hemorrhagic events (*P* > 0.999), postoperative hospital stay (*P* = 0.715), as well as functional outcome at discharge (*P* = 0.332) between the two groups.

**Conclusions:**

Our preliminary results showed that long-term low-dose aspirin use was not associated with worse outcomes. Early surgery can be safe for ruptured intracranial aneurysms in patients with long-term aspirin use.

## Background

Acetylsalicylic acid (aspirin) is the most widely used antiplatelet drug in primary prevention and secondary prevention of atherosclerosis diseases [1]. Chronic use of low-dose aspirin can significantly inhibit platelet aggregation. Past researches have studied the effects of preoperative aspirin use on bleeding risk of noncardiac surgeries. Large studies reported an increased risk of bleeding complications in patients with continuation of preoperative aspirin undergoing noncardiac surgeries [2-4], while some studies believed that perioperative discontinuation of aspirin raised the risk of cardiovascular accident without lowering bleeding risk [5, 6]. However, these studies seldom included patients undergoing craniotomy operations. Because of the serious consequences of intracranial hemorrhage, many surgeons suggested aspirin therapy be stopped for more than 7 days before elective neurosurgery to avoid the increased bleeding risk [7, 8]. However, this option does not apply to emergency situation. Aneurysm rebleeding is associated with significant mortality and poor outcomes after intracranial aneurysmal subarachnoid hemorrhage (aSAH) [9]. Thus, the recommended timing to repair ruptured aneurysms has become early within 72 h after the onset of aSAH [10-12]. However, dilemma frequently occurs when managing patients with current aspirin use. The association between aspirin use and postoperative outcomes in aSAH patients undergoing early surgery remains unclear. A recent traumatic neurosurgical study found that low-dose aspirin use without preoperative cessation was not associated with unfavorable outcomes after emergency surgery for traumatic intracranial hemorrhage [13]. Further evidence on the safety of early surgery in aSAH patients with long-term aspirin use is needed.

In this study, we reviewed a consecutive series of patients with and without aspirin use who underwent early microsurgical clipping for aSAH. We compared perioperative bleeding events and postoperative outcomes between aspirin and non-aspirin group, aiming to examine whether aspirin use was associated with worse outcomes after early surgery for ruptured aneurysms.

## Methods

### Patient selection

This retrospective study was approved by the Institutional Review Board. The procedures followed were in accordance with the ethical standards with the Helsinki Declaration. Between 2012 and 2018, a total of 215 patients underwent early microsurgery after intracranial aneurysm rupture in our institution. We included the patients with the following inclusion criteria: (1) SAH confirmed by plain computed tomography (CT), with responsible aneurysms detected by CT angiography (CTA) or digital subtraction angiography (DSA), (2) treated by microsurgical clipping within 72 h after aSAH onset. The exclusion criteria used were (1) non-aneurysmal subarachnoid hemorrhage, (2) multiple aneurysms treated simultaneously, (3) undergoing operation other than direct clipping (e.g., wrapping or trapping), (4) currently using clopidogrel, warfarin, and other antithrombotic drugs before surgery.

In our institution, aSAH patients with Hunt-Hess (HH) grade ≤ 3 are commonly treated early. Aneurysms located in internal carotid artery or posterior circulation are firstly treated by endovascular treatment, while distal aneurysms, aneurysms not suitable for coiling, and aSAH combined with large hematoma or brain hernia are considered to be treated by microsurgery. The preference of patient or family influenced treatment decision as well.

### Data collection

Demographic, aneurysm, and clinical data were retrospectively collected from our electronic medical database. Patient characteristics included sex, age, the dosage of preoperative aspirin use, and any history of the following comorbidities: cardio-cerebrovascular accident, hypertension, or diabetes mellitus. Aneurysm characteristics included size and site. The maximum diameter of aneurysm was measured on preoperative three-dimensional CTA or DSA. The site was roughly divided into anterior circulation and posterior circulation aneurysms. Clinical data collected included admission HH grade, modified CT Fisher grade [14], intraoperative blood loss, postoperative hemorrhage events, and neurological function at discharge. The HH grade and modified Fisher scale were assessed by two experienced investigators based on preoperative clinical condition and brain CT scan. The HH grade was divided into two groups: grades 1 to 3 and grades 4–5. The modified Fisher grade was classified as grade 1, grades 2–3, and grade 4 [14]. The history of taking antiplatelet drugs was recorded. Patients were divided into aspirin group and non-aspirin group according to whether they received chronic aspirin therapy or not.

### Outcome assessment

The outcomes assessed in this study were as follows: (1) intraoperative blood loss, (2) postoperative hemorrhagic events, (3) postoperative infarction, (4) hospital mortality, (5) length of postoperative hospital stay, and (6) functional outcome at discharge.

Intraoperative blood loss was collected from the anesthesia record. Postoperative hemorrhagic events included intracranial and gastrointestinal bleeding. Intracranial hemorrhage or cerebral infarction was confirmed by postoperative CT scan. Hemorrhage from aneurysm intraoperative rupture or postoperative rebleeding caused by unsuccessful clipping was excluded. Neurological function was evaluated with the modified Rankin Scale (mRS). Functional impairments clearly associated with other comorbidities were not ascribed to surgery. An mRS score ≥ 3 meant an unfavorable outcome.

### Statistical analysis

We performed statistical analysis by using SPSS 22.0 (SPSS Inc., Chicago, IL, USA). Continuous variables are presented as mean ± standard deviation (SD) and categorical variables as number (%). To minimize confounding bias associated with nonrandomized retrospective design, we did propensity score matching (PSM) to balance the baseline characteristics of the aspirin and non-aspirin group. The propensity score of each patient was estimated with logistic regression model. The following potential confounding factors were used as covariates: sex, age, comorbidity history, admission HH grade, CT modified Fisher grade, aneurysm size, and site. The matching rate was 1:2 for the aspirin group to non-aspirin group, and the caliper width was set at 0.2. The nearest neighbor method was used. To test the calibration effectiveness of the PSM, the baseline characteristics were compared between the aspirin and non-aspirin group before and after the 1:2 matching. Then, we compared the outcomes between the two matched groups. We used Chi-square test to compare categorical variables. Continuous variables were compared by Mann-Whitney *U* test. The level of significance was set at *P* < 0.05, and all tests were 2-sided.

## Results

### Demographics and clinical characteristics

After data cleansing, 215 patients undergoing early microsurgery for ruptured intracranial aneurysms were reviewed. Among them, 21 patients were classified into aspirin group and 194 into non-aspirin group, according to preoperative aspirin use. The mean interval from onset to surgery was 2.5 ± 0.6 days. Twenty patients in the aspirin group received 100 mg aspirin per day, and 1 patient had a dosage of 75 mg per day. Aspirin was stopped immediately after aSAH onset. The baseline characteristics of the two groups are showed in Table 1. Because of the objective selection bias, patients in the aspirin group had an older mean age (*P* = 0.037) as well as a higher rate of past comorbidity history (*P* = 0.001), compared with those in the non-aspirin group. After the 1:2 PSM, 42 matched patients were extracted from the non-aspirin group. The aspirin group consisted of 14 females and 7 males with a mean age of 58.9 ± 8.4 years. The mean aneurysm size was 6.3 ± 2.6 mm, and the mean admission modified CT fisher grade was 3.2 ± 1.1. The non-aspirin group consisted of 32 females and 10 males with a mean age of 57.2 ± 10.3 years. The mean aneurysm size was 6.5 ± 3.2 mm, and the mean admission modified CT fisher grade was 3.2 ± 1.0. Comparison between these two matched groups showed no significant difference in demographic, aneurysm, and clinical baseline characteristics (Table 1).

**Table 1:** Characteristics of patients with and without long-term aspirin use before and after matching

Characteristics	Before matching			After matching		
	Aspirin group (*n* = 21)	Non-aspirin group (*n* = 194)	*P* value	Aspirin group (*n* = 21)	Non-aspirin group (*n* = 42)	*P* value
Age (year)	58.9 ± 8.4	53.9 ± 10.4	0.037	58.9 ± 8.4	57.2 ± 10.3	0.737
Sex			0.666			0.422
Male	7 (33.3)	74 (38.1)		7 (33.3)	10 (23.8)	
Female	14 (66.7)	120 (61.9)		14 (66.7)	32 (76.2)	
Hunt-Hess grade			> 0.999			> 0.999
1–3	20 (95.2)	186 (95.9)		20 (95.2)	40 (95.2)	
4–5	1 (4.8)	8 (4.1)		1 (4.8)	2 (4.8)	
Modified Fisher grade			0.331			0.285
1	3 (14.3)	13 (6.7)		3 (14.3)	2 (4.8)	
2–3	5 (23.8)	68 (35.1)		5 (23.8)	16 (38.1)	
4	13 (61.9)	113 (58.2)		13 (61.9)	24 (57.1)	
Aneurysm size (mm)	6.3 ± 2.6	6.2 ± 3.3	0.421	6.3 ± 2.6	6.5 ± 3.2	0.901
Site			> 0.999			> 0.999
Anterior circulation	21 (100)	191 (98.5)		21 (100)	42 (100)	
Posterior circulation	0	3 (1.5)		0	0	
Comorbidity history	20 (95.2)	113 (58.2)	0.001	20 (95.2)	42 (100)	0.333
Mean propensity score	0.173	0.090		0.165	0.163	

Categorical variables are presented as number (%), and continuous variables are presented as mean ± standard deviation

### Treatment outcomes

**Fig. 1:**
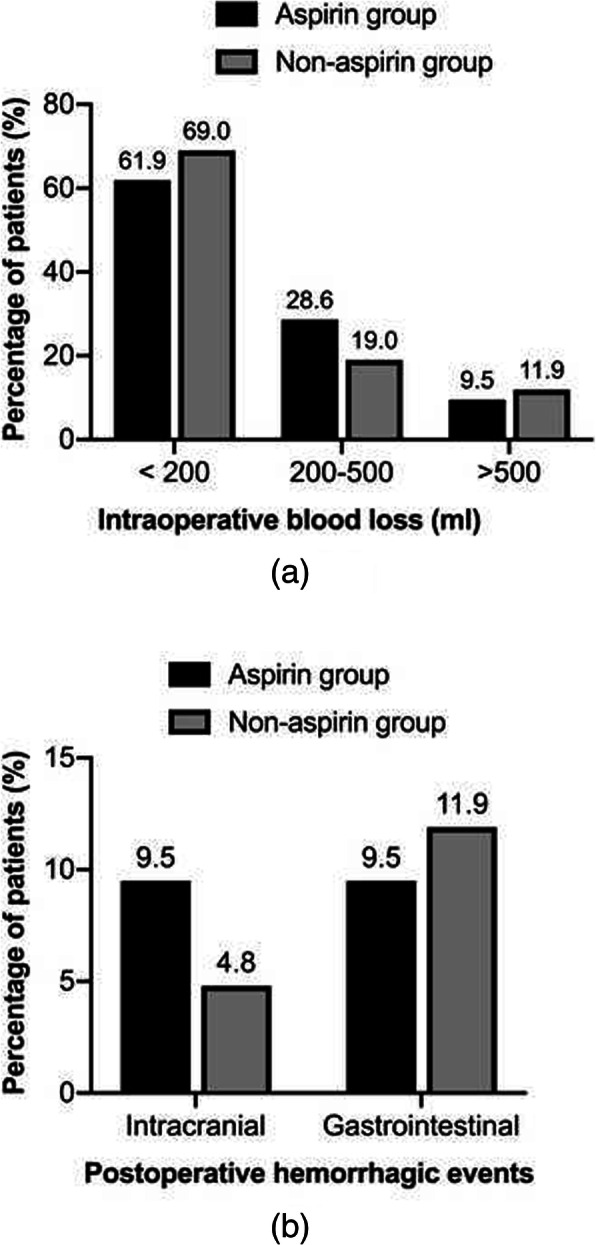
Bleeding events associated with the aspirin group and non-aspirin group: the rate of patients according to intraoperative blood loss (**a**), and the incidence of postoperative hemorrhagic events (**b**)

None of the 63 patients received blood product transfusion before surgery. Table 2 summarizes the treatment outcomes of the two matched groups. The mean intraoperative blood loss of the aspirin group was 257.1 ± 119.7 ml, while that of the non-aspirin group was 296.4 ± 280 ml with no significant difference (*P* = 0.540). In regard to postoperative complications, the rate of hemorrhagic events in the aspirin group (19.0%) was higher than that in the non-aspirin group (16.7%), but this did not reach statistical significance (*P* > 0.999). Figure 1 compares the rate of patients according to intraoperative blood loss and the incidence of postoperative hemorrhagic events between the two groups. Postoperative cerebral infarction occurred in 6 patients in the aspirin group with the rate of 28.6%, which is similar with that (31.0%) in the non-aspirin group (*P* = 0.846). No patient died during hospitalization. The mean length of postoperative hospital stay was 14.0 ± 5.4 and 14.5 ± 5.6 days respectively in the two groups. In regard to the functional status at discharge, favorable outcome was obtained in 13 of 21 (61.9%) patients in the aspirin group, compared with 31 of 42 (73.8%) patients in the non-aspirin group. However, this did not reach statistical significance (*P* = 0.332).

**Table 2:** Comparison of postoperative outcomes between aspirin and non-aspirin group

Outcomes	Aspirin group (*n* = 21)	Non-aspirin group (*n* = 42)	*P* value
Intraoperative blood loss (ml)	257.1 ± 119.7	296.4 ± 280.8	0.540
Postoperative hemorrhagic events	4 (19.0)	7 (16.7)	> 0.999
Postoperative cerebral infarction	6 (28.6)	13 (31.0)	0.846
Postoperative hospital stay (days)	14.0 ± 5.4	14.5 ± 5.6	0.715
Functional outcome			0.332
mRS < 3	13 (61.9)	31 (73.8)	
mRS ≥ 3	8 (38.1)	11 (26.2)	

Categorical variables are presented as number (%), and continuous variables are presented as mean ± standard deviation

## Discussion

Low-dose aspirin has been used to prevent cardio-cerebrovascular diseases by a wide range of population [1], including some emergency aSAH patients. In the consecutive series of this study, 9.8% of patients were taking long-term aspirin before the onset of aSAH. This incidence is consistent with the rate of 9.5 to 11.7% reported in past studies [15, 16]. The influence of prehemorrhage aspirin use on the presenting severity has been examined. Gross et al. [16] found that neither HH grade nor Fisher grade was associated with aspirin use. Dasenbrock et al. [17] even reported a better NIS-SAH severity score in patients taking aspirin. In the present study, we did not observe significant differences in aSAH presenting severity between patients with and without aspirin use, which coincided with the former research. Notably, Toussaint et al. [15] reported increased rebleeding rates in aspirin users, which could be explained by the antiplatelet effect of aspirin.

Rebleeding is the common cause of significantly poor outcome after aSAH, which can occur until definite treatment of ruptured aneurysms. During the past decades, early treatment within 72 h after onset of aSAH has been recommended to improve outcomes. However, because of the increased bleeding risk associated with antiplatelet agents, the safety of early surgery for aSAH patients receiving aspirin has been overshadowed. In this study, we evaluated the relationships between preoperative aspirin use and outcomes after early microsurgical clipping. Although patients with aspirin use were significantly older and had a greater rate of comorbidities than those without, after propensity score matching, we found no differences in intraoperative blood loss, postoperative hemorrhagic events, cerebral infarction, length of hospital stay, as well as unfavorable functional outcome between the aspirin group and non-aspirin group. These results suggested that long-term low-dose aspirin use was not associated with adverse outcomes after early surgery of aSAH. This is in agreement with the findings of a recent traumatic neurosurgical study. In that retrospective study involving 171 patients (87 receiving preoperative aspirin), they concluded that low-dose aspirin use without preoperative cessation was not associated with unfavorable outcomes after emergency surgery for traumatic intracranial hemorrhage [13].

Nevertheless, published literatures did not reach a consensus on the relationships between aspirin use and neurosurgical outcomes. In another traumatic study with small sample size, a decreased mortality was noted in aspirin users with traumatic subdural hematoma [18]. By contrast, Li et al. [19] examined the effect of preoperative aspirin use on outcomes of patients with spontaneous intracranial hemorrhage undergoing craniotomy, finding significantly increased rates of intraoperative blood loss, postoperative hemorrhage, and mortality in aspirin users compared with non-users. Few literatures have focused on the association between aspirin use and postoperative outcomes in aSAH patients undergoing microsurgical clipping. Dasenbrock et al. [17] found that neither hospital mortality nor total complication rates was associated with long-term aspirin use in aSAH patients treated surgically, but they did not note the time interval to surgery or whether aspirin was discontinued preoperatively.

In the present study, we evaluated the safety of early surgery with preoperative interval less than 3 days in aSAH patients with long-term aspirin use, finding no greater risks was associated with these patients. Aneurysm clipping is an “out of brain” operation, of which microsurgical maneuvers are commonly done in subarachnoid space rather than parenchyma. The possibility of intraoperative or postoperative tissue wound bleeding was much lower, compared with traumatic or spontaneous intracranial hemorrhage. The serious danger of aSAH surgery mainly comes from intraoperative aneurysm rebleeding. Thus, the safety of aSAH for aspirin users could be theoretically explained. We suggest early surgery for aSAH patients despite taking long-term aspirin mainly to eliminate the rebleeding risk. Besides, early surgery means a shorter duration of aspirin discontinuation, which can bring potential benefits. Previous research revealed that preoperative withdrawal of aspirin could significantly increase thrombotic risks, especially for patients with artery stents [3, 20]. In our series, 14 patients of the aspirin group had history of cardio-cerebrovascular accidents. In our institution, for patients with long-term aspirin use, antiplatelet is suggested to be early restarted after 24 h postoperatively if no hemorrhagic events occur.

Platelet transfusion has been recommended as a direct method to reverse the antiplatelet effect of aspirin in patients with intracranial hemorrhage who will undergo neurosurgery [21]. Li et al. [19] found significantly improved outcomes after intracranial hematoma evacuation in patients with aspirin use who received preoperative platelet transfusion. Controversially, evidence against platelet transfusion was reported by the PATCH trial [22]. Although in the present study positive outcomes were obtained in patients of the aspirin group without preoperative platelet transfusion, in our opinion, reversal of aspirin should be considered when platelet function is severely inhibited or aSAH is complicated with large hematoma.

### Limitation

This study has some limitations. First, this is a small retrospective cohort study with selection bias. Although confounding factors have been corrected by the propensity score matching design, prospective controlled studies with sufficient population are needed to provide conclusive evidence. Second, data of platelet function testing or thromboelastography was lacking. Possible aspirin resistance might interfere with the results. Third, aSAH patients undergoing endovascular treatment were not studied in the present work. Despite these limitations, this study is the first to evaluate the safety of early surgery for ruptured intracranial aneurysms in patients with long-term aspirin use, providing preliminary evidence for decision-making.

## Conclusions

Our preliminary results showed that long-term low-dose aspirin use was not associated with increased intraoperative blood loss, postoperative hemorrhagic events, length of hospital stay, as well as unfavorable functional outcome. Early surgery can be safe for ruptured intracranial aneurysms in patients with long-term aspirin use.

## Data Availability

Please contact author for data request.

## Abbreviations

aSAH: Aneurysmal subarachnoid hemorrhage
PSM: Propensity score matching
